# Character Strengths in the Life Domains of Work, Education, Leisure, and Relationships and Their Associations With Flourishing

**DOI:** 10.3389/fpsyg.2021.597534

**Published:** 2021-04-21

**Authors:** Lisa Wagner, Lisa Pindeus, Willibald Ruch

**Affiliations:** Department of Psychology, University of Zurich, Zurich, Switzerland

**Keywords:** character strengths, strengths-related behavior, applicability of character strengths, strengths use, strengths deployment, life domains, flourishing, well-being

## Abstract

A growing body of research demonstrates the relevance of character strengths for flourishing in general, but also for important outcomes across different life domains (e.g., work performance and relationship satisfaction). Studies have also shown that there are differences in the extent to which character strengths are applied, that is, perceived as relevant and shown in behavior in a given context, between work and private life, but they have not considered other life domains. This study aims to close this gap by examining the life domains of work, education, leisure, close personal relationships, and romantic relationships. The present study investigates whether (a) strengths-related behavior across different life domains explains additional variance in flourishing beyond the trait level of each respective character strength and studies (b) differences in the relevance of character strengths and strengths-related behavior across different life domains, and examines (c) their relationships with flourishing. A sample of 203 German-speaking adults (78.8% females; mean age = 29.4 years) completed self-reports assessing flourishing and character strengths. They also indicated which of the five life domains were personally relevant to them (i.e., on average 4.23 life domains) and reported the character strengths' perceived relevance and the frequency of displaying strengths-related behavior for each of these life domains separately. The results demonstrate that (a) strengths-related behavior averaged across all relevant life domains explained unique variance in flourishing above the trait-level of character strengths in some cases (e.g., creativity, kindness, and fairness), (b) different life domains were characterized by specific profiles of character strength—regarding both their relevance and strength-related behavior. Moreover, (c) character strengths and strengths-related behavior in different life domains both showed substantial correlations with flourishing. In some cases, these associations were domain-specific (e.g., displaying love of learning in the context of education was related to higher levels of flourishing). In conclusion, we suggest that examining strengths-related behavior across different life domains represents a worthwhile addition to research on character strengths.

## Introduction

Do we experience flourishing when we are creative in our leisure time? Is love of learning displayed particularly often in the context of education? Do we consider prudence to be more relevant at work than in other domains of life? Character strengths, such as creativity, love of learning, and prudence, are conceptualized as positively valued personality traits (Peterson and Seligman, 2004), so we generally assume that they can be and are displayed across a variety of different situations in various life domains (see Niemiec, 2020). Character strengths can be investigated at different levels. Typically, they are conceptualized as traits that are relatively stable across time and context. However, investigating whether character strengths are perceived as important and displayed in a specific life domain allows for a more nuanced understanding of the role character strengths play in different life domains.

Empirical findings demonstrate the relevance of character strengths both for well-being and flourishing in general (e.g., Hausler et al., 2017a; Wagner et al., 2020a) and for desirable outcomes in different life domains (e.g., workplace, Harzer and Ruch, 2014; Heintz and Ruch, 2020, or education, Lounsbury et al., 2009; Wagner and Ruch, 2015). These findings suggest that some character strengths are relevant across all life domains, but some strengths might be of particular relevance to specific life domains (e.g., love of learning to education). The present study aims to extend the knowledge on the role of character strengths across different life domains. To achieve this aim, we assessed character strengths as traits (i.e., as individual differences that are relatively stable across time and context) as well as the character strengths' relevance and strengths-related behavior (i.e., perceived importance of each respective character strength and the frequency with which one displays behavior consistent with that character strength) for each life domain. Specifically, we investigated whether (a) strengths-related behavior across different life domains explained additional variance in flourishing beyond the contribution of character strengths as traits, (b) life domains differed concerning the perceived relevance of character strengths as well as the frequency of their display, and (c) perceived relevance and strengths-related behavior across life domains were related to a global assessment of flourishing.

### Character Strengths

The Values in Action (VIA) classification (Peterson and Seligman, 2004) describes character strengths as a family of positively valued traits, a set of qualities that enable individuals (and their communities) to thrive, that is, to achieve optimal psychological functioning or flourishing. The classification represents a cornerstone of positive psychology, which is aimed at studying what makes life worth living (Seligman and Csikszentmihalyi, 2000). The VIA classification comprises 24 character strengths that are assigned to six core virtues: creativity, curiosity, judgment, love of learning, and perspective (assigned to the virtue of wisdom and knowledge); bravery, perseverance, honesty, and zest (assigned to the virtue of courage); love, kindness, and social intelligence (assigned to the virtue of humanity); teamwork, fairness, and leadership (assigned to the virtue of justice); forgiveness, humility, prudence, and self-regulation (assigned to the virtue of temperance); and appreciation of beauty and excellence gratitude, hope, humor, and spirituality (assigned to the virtue of transcendence).

These character strengths were selected based on a broad review of positively valued traits in research, history, and popular culture across different cultures (Peterson and Seligman, 2004) and had to fulfill most of the 12 criteria to be included (see Ruch and Stahlmann, 2019). One of these criteria is that character strengths are trait-like characteristics, which demonstrate relative stability across time and different situations. Relative stability means that traits are shown to a similar degree across situations, but there is also variability between different contexts. Variability in the enactment of personality traits across situations has many sources (e.g., Green et al., 2019), yet it can be argued that life domains account for a relatively large part of this variability, in part because they vary in goals and social roles to be fulfilled (see, e.g., Bleidorn and Denissen, 2015). Character strengths are thus expected to be displayed across all life domains of an individual, yet to also show variation across life domains. Harzer and Ruch (2013) have demonstrated this for the broad distinction between work and private life; the present study takes a closer look at this by studying life domains in more detail.

### Character Strengths and Flourishing

Previous studies have provided consistent support for the relationship of the 24 character strengths described in the VIA classification with various facets of well-being and flourishing (e.g., Peterson et al., 2007; Proyer et al., 2011, 2013; Buschor et al., 2013; Martínez-Martí and Ruch, 2014; Hausler et al., 2017a; Gander et al., 2020c; Wagner et al., 2020a). The character strengths of curiosity, zest, love, gratitude, and hope have consistently shown the most substantial relationships with subjective well-being. In addition to this set of strengths, the character strengths of honesty, perseverance, kindness, social intelligence, self-regulation, and humor have been found to be robustly related to overall psychological well-being (Hausler et al., 2017a). However, there were also hints at differential relationships of character strengths with specific aspects of well-being, such as mastery or accomplishment in the case of perseverance (Hausler et al., 2017a; Wagner et al., 2020a), giving rise to the idea that character strengths contribute differentially to various life outcomes and as a consequence may vary in their relevance across life domains.

Variations between contexts can be studied by investigating the display of character strengths across different situations (i.e., in various life domains). This has been done using varying terminologies—for example, “application,” “applicability,” “use,” “deployment,” or “strengths-related behavior”—which all refer to the extent to which a person shows behavior related to a character strength in a given context. However, the term “applicability,” character strengths as introduced by Harzer and Ruch (2012, 2013), covers four aspects: (a) the promotion (“it is encouraged”), (b) the helpfulness (“it is helpful”), and (c) the importance (“it is important to me”) of a character strength as well as (d) strength-related behavior (“I behave like this”) in the respective context. By taking the aspects of promotion, helpfulness, and importance into account, this conceptualization specifically acknowledges the role of environmental demands that might influence the degree to which a character strength can be displayed in a given context—in other words, the character strengths' relevance in the context.

Typically, all four aspects assessed by the Applicability of Character Strengths Rating Scales (ACS-RS; Harzer and Ruch, 2013) are summed up into a total score. In the present study, however, we considered the perceived relevance in a given context (i.e., the items referring to promotion, helpfulness, and importance) separately from the display of strengths-related behavior (i.e., the item assessing behavior) to provide a more nuanced picture of the relationships studied. The item assessing strengths-related behavior is highly similar to other assessments of strengths deployment, which is displaying character strengths in a given context, such as the Strengths Deployment Measure (Littman-Ovadia and Steger, 2010; Littman-Ovadia et al., 2017). While both aspects (relevance and strengths-related behavior) reflect an individual's perception, we argue that perceived relevance more strongly refers to features of the context, whereas strengths-related behavior refers directly to the display of behavior.

Overall, it has been assumed that the display of character strengths is related to individual well-being, and that individual well-being can be increased by displaying character strengths (Peterson and Seligman, 2004). Randomized placebo-controlled trials have indeed shown that the positive psychological interventions that instruct participants to find new ways to display their signature strengths (i.e., those strengths that are most typical of an individual) are effective in improving well-being and alleviating depressive symptoms (see Schutte and Malouff, 2019, for a meta-analysis).

However, it is unclear whether the effectiveness of such interventions is limited to increasing the display of signature strengths—two studies found that the same intervention was equally effective when it was not limited to an individual's signature strengths (Rust et al., 2009; Proyer et al., 2015). Therefore, it seems that displaying character strengths is generally beneficial for well-being, irrespective of whether the strengths are the individual's signature strengths. In addition, the VIA Inventory of Strengths (VIA-IS; Peterson and Seligman, 2004) is not designed to assess signature strengths, and it has not been tested whether the five highest strengths in the VIA-IS match the signature strengths assessed by different means (i.e., *via* interview or by testing the proposed criteria for signature strengths directly; Ruch, 2013). As a consequence, the present study investigated the role of displaying all character strengths across various life domains, without limiting the focus to signature strengths.

Huber et al.'s (2020) results provided the first support for the idea that the applicability of a character strength might explain additional variance in well-being beyond the influence of the trait level of the respective strength. However, the authors only addressed this question for five of the 24 character strengths and in smaller subsamples of *n* < 100. In the present study, we investigate this question for all character strengths and also extend the life domains studied.

Building on the results reviewed, we derived the following expectations:

Hypotheses 1.1–1.11: We expect the character strengths of curiosity, perseverance, honesty, zest, love, kindness, social intelligence, self-regulation, gratitude, hope, and humor to be positively related to flourishing.

Hypotheses 2.1–2.11: We expect the mean strengths-related behavior across life domains for the character strengths of curiosity, perseverance, honesty, zest, love, kindness, social intelligence, self-regulation, gratitude, hope, and humor to be positively related to flourishing.

Hypothesis 3: We expect the aggregat level of strengths-related behavior across different life domains to explain unique variance in flourishing when analyzed together with the VIA-IS scores of all 24 character strengths.

### Character Strengths' Roles Across Different Life Domains

The first criterion for a character strength is that it contributes to different fulfillments that make up a “good life” (Peterson and Seligman, 2004). Previous research has shown that character strengths differentially relate to orientations to well-being (Seligman, 2011; pleasure/positive emotions, engagement, meaning, and accomplishment; Wagner et al., 2020a). It can be assumed that different life domains offer different opportunities for fulfillment, and thus, it can be assumed that the character strengths' relevance varies between life domains, at least to a certain extent. While previous studies only considered one life domain or compared the broad domains of private and work life, the present study focuses on character strengths in a range of different life domains that we consider most relevant in the lives of (young) adults: work, education, leisure, close personal relationships, and romantic relationships.

In the following, we provide an overview of the evidence regarding associations between (a) character strengths and (b) the applicability of character strengths or strengths-related behavior with relevant outcomes in the respective life domain, as well as any studies providing information on (c) the perceived relevance of character strengths in this context, for each of these five life domains. From these findings, we derive hypotheses on which character strengths are of particular relevance in each respective life domain. Given the scarcity of results differentiating (a), (b), and (c), we tentatively assume that the character strengths of particular relevance are (1) perceived as more relevant and (2) displayed more frequently in behavior than in other life domains and that their (3) relevance and (4) display in this life domain are positively related to flourishing. It is, however, likely that the results regarding these aspects will diverge for some of the character strengths.

#### Work

Much research has focused on the role of character strengths in the workplace. In particular, the character strengths of zest and perseverance have been highlighted as particularly conducive to work-related outcomes, including being satisfied with one's work, perceiving one's work as meaningful, showing little counterproductive work behavior, and performing well (Peterson et al., 2009; Littman-Ovadia and Lavy, 2016).

While the character strengths associated most strongly with job satisfaction overlap with those most strongly associated with life satisfaction (i.e., zest, hope, curiosity, love, and gratitude), differential relationships were also found for different occupational subgroups (Peterson et al., 2010; Heintz and Ruch, 2020). Gander et al. (2020b) recently showed in a sample that was nationally representative for Switzerland that work satisfaction is not only concurrently but also predictively related to character strengths (i.e., zest, love, kindness, social intelligence, leadership, forgiveness, gratitude, and hope showed positive relationships at all time points). Underlining the role of the work context, a fit with the typical character strengths' configuration in the occupational group is also relevant for job satisfaction (Peterson et al., 2010; Gander et al., 2020b). Gander et al. (2012) found that character strengths explained 35% of the variance in satisfaction with work results (as compared with 53% of the variance in life satisfaction), with the character strengths of hope, perseverance, zest, curiosity, perspective, and bravery yielding the highest correlations. Character strengths have also been shown to relate to adaptive coping strategies in the workplace and lower levels of work-related stress or burnout (Harzer and Ruch, 2015; Allan et al., 2017). Further, some character strengths go along with better self- and supervisor-rated work performance (Harzer and Ruch, 2014; Gander et al., 2020a), including both task performance and contextual performance. Finally, differences in character strengths have been observed in working versus retired individuals after controlling for age in a sample representing middle adulthood to old age, although the observed effects were small (Baumann et al., 2020).

The role of displaying character strengths in the context of work has also been studied. For instance, Littman-Ovadia and Steger (2010) presented the names of the 24 character strengths of the VIA classification and asked employees and volunteers to rate the extent to which they had the opportunity to display the respective strengths in their daily activities, which was then summed up into an overall score. This global assessment was positively related to overall well-being and meaning (in both life and work) across both groups. A more nuanced approach for the assessment of displaying character strengths at work was suggested by Harzer and Ruch (2012, 2013, 2014): using the ACS-RS, they showed that (1) there are differences in the applicability of character strengths between private and work life (e.g., all character strengths assigned to the virtues of wisdom and knowledge were more applicable in work life, whereas all character strengths assigned to the virtues of courage and humanity were more applicable in private life), (2) the relationship between an individual's level of a character strength score and the applicability was, on average, of medium and similar size for both work and private life (i.e., there seemed to be a similar degree of environment selection in both contexts), and (3) the applicability of character strengths at work (also when rated by supervisors) was positively related to both well-being and performance at work. These findings were corroborated by a randomized, placebo-controlled intervention that found increases in calling and life satisfaction for the intervention group that was instructed to show their four highest character strengths more frequently at work for 4 weeks in comparison with the control group that was instructed to reflect on four situations in which they were “at their best” (Harzer and Ruch, 2016).

Extending this work, several studies have been focused on the applicability of signature character strengths (typically the four character strengths in which an individual scores highest) at the workplace and its relationship to work-related and general well-being (Hausler et al., 2017b; Merritt et al., 2019; Höge et al., 2020; Huber et al., 2020; Strecker et al., 2020). Among these, Huber et al. (2020) also report initial results suggesting that, in particular, the applicability of judgment at work is relevant to work-related outcomes, such as higher levels of work engagement and lower levels of emotional exhaustion and depersonalization. Another group of studies has used generic measures of strengths use that are not related to the character strengths of the VIA classification (e.g., Dubreuil et al., 2014; Lavy and Littman-Ovadia, 2016; Bakker and van Woerkom, 2018). The studies converge in supporting the notion that showing strengths-related behavior at work is conducive to desirable outcomes; however, given the general nature of the assessment of strengths use (using the phrase “my strengths,” which can be construed in very different ways by participants), their results are of limited usefulness for the present study. Nonetheless, these studies support the claim that strengths-related behavior at work is relevant for both work-related and global life outcomes.

Hypotheses 4.1–4.6: Building on the results reviewed, we expect the character strengths of judgment, perseverance, zest, teamwork, leadership, and self-regulation to be of particular relevance in the life domain of work (i.e., that they will be perceived as more relevant and displayed more frequently in behavior than in other life domains and that their relevance and display in this life domain will be positively related to flourishing).

#### Education

The role of character strengths in educational contexts has mostly been studied in adolescents. Studies demonstrate that (a) several character strengths are related to educational outcomes, including school-related well-being, positive classroom behavior, and school achievement; (b) these associations are robust when controlling for the influence of cognitive ability; and (c) there is a differential pattern of associations depending on the outcome of interest (e.g., prudence and self-regulation seem to be of particular relevance for positive classroom behavior), but certain character strengths (in particular, perseverance and love of learning) seem to be of general relevance in the educational setting (e.g., Park and Peterson, 2006; Weber and Ruch, 2012a; Wagner and Ruch, 2015, 2021; Weber et al., 2016; Wagner et al., 2020b).

Wagner and Ruch (2021) studied the role of strengths-related behavior at school for academic achievement and school-related well-being. Drawn from a diary study, the results demonstrate that strengths-related behavior in the context of school explained additional variance in educational outcomes (school achievement and well-being) beyond the level of the character strengths (i.e., the “possession” of the respective strength), on both the between-person and the within-person level. This study's findings also substantiate the notion of separating the perceived relevance of character strengths from the display of strengths-related behavior in a given context: several character strengths were perceived as highly relevant at school but were not reported to be shown frequently (e.g., love of learning and leadership), whereas other character strengths (e.g., humility and humor) were not perceived as highly relevant but shown frequently.

In the context of college or university education, perseverance has emerged as a consistent predictor of academic achievement (as assessed by grade point average), and college satisfaction was found to be highly correlated with the character strengths of hope, self-regulation, zest, and perseverance (Lounsbury et al., 2009; Karris Bachik et al., 2020). In addition, love of learning has also been found to relate to the educational level obtained in adults, hinting at its general relevance for education (Ruch et al., 2010).

Kachel et al. (2020) investigated the applicability of signature character strengths in studies and private life in a sample of medical students. Overall, they found higher scores for the applicability of signature character strengths in private life than for the applicability in university life, in particular for those students with high or increasing levels of cynicism. While this suggests that applying signature strengths during studies may be related to higher levels of well-being, because of the methodological approach of only assessing the applicability of each individual's five highest character strengths, these results do not advance the question of which character strengths are most relevant in the university context.

Hypotheses 5.1–5.4: Building on the results reviewed, we expect the character strengths of love of learning, perseverance, prudence, and self-regulation to be of particular relevance in the life domain of education (i.e., that they will be perceived as more relevant and displayed more frequently in behavior than in other life domains and that their relevance and display in this life domain will be positively related to flourishing).

#### Leisure

To date, very little research has considered the role of character strengths in leisure activities. Satisfaction with leisure time assessed globally was found to relate positively to curiosity, zest, love, gratitude, hope, and humor (Ruch et al., 2010)—a set of character strengths almost identical to those that consistently show the highest correlations with life satisfaction (e.g., Buschor et al., 2013), so this finding might not be specific to leisure activities *per se*. In a qualitative study regarding a very specific leisure activity, participants in charity sports events indicated showing zest, kindness, teamwork, and hope during these events (Coghlan and Filo, 2016). We expect character strengths' relevance to differ between different leisure activities (as it differs between different occupations or even workplaces). However, certain character strengths, such as curiosity, love of learning, and appreciation of beauty and excellence, might be more commonly relevant in leisure activities that involve cultural activities (see Ruch et al., 2010), whereas the character strength of zest might facilitate the initiation of leisure activities in general. It can also be assumed that leisure time offers more opportunities than other life domains to display creativity and spirituality and, as a consequence, that these character strengths are perceived as more relevant in this context.

Hypotheses 6.1–6.6: Building on the results reviewed, we expect that the character strengths of creativity, curiosity, love of learning, zest, appreciation of beauty and excellence, and spirituality would be of particular relevance in the life domain of leisure (i.e., that they will be perceived as more relevant and displayed more frequently in behavior than in other life domains and that their relevance and display in this life domain will be positively related to flourishing).

#### Close Personal Relationships

We use the term “close personal relationships” to describe intimate relationships with family and friends. An orientation to positive relationships (i.e., to having close personal relationships) was found to be consistently related to the character strengths of honesty, zest, love, kindness, social intelligence, teamwork, fairness, leadership, forgiveness, humility, gratitude, and humor across different samples and self- and informant ratings (Wagner et al., 2020a). A strongly overlapping set of character strengths (curiosity, honesty, zest, love, kindness, social intelligence, teamwork, fairness, leadership, gratitude, hope, and humor) correlated positively with satisfaction with friendships, and kindness, social intelligence, and humor were additionally correlated with spending more time with friends during a typical month (Ruch et al., 2010). In a sample of adolescents, the character strengths of perspective, honesty, love, kindness, social intelligence, teamwork, leadership, gratitude, and humor were identified as most relevant for positive peer relationships across several analyses (desired characteristics in a friend, associations with likeability, number of friends, and friendship quality and satisfaction; Wagner, 2019).

Strengths-related behavior in the context of close personal relationships has been shown to relate to mood regulation: in their quasi-experimental diary study, Lavy et al. (2014) found that unfavorable mood enhanced strengths-related behavior on the following day. Conversely, strengths-related behavior was related to higher levels of positive daily mood on the following day, and this effect was stronger in the experimental group, in which participants were instructed to write a note to a loved person every day. These results suggest that close personal relationships increase the positive consequences of strengths-related behavior. However, no study in the context of close personal relationships has considered strengths-related behavior at the level of character strengths.

Hypotheses 7.1–7.10: Building on the results reviewed, we expect the character strengths of honesty, love, kindness, social intelligence, teamwork, fairness, leadership, forgiveness, gratitude, and humor to be of particular relevance in the life domain of close personal relationships (i.e., that they will be perceived as more relevant and displayed more frequently in behavior than in other life domains and that their relevance and display in this life domain will be positively related to flourishing).

#### Romantic Relationships

Individuals who are currently in romantic relationships or cohabitating with a partner report a different trait levels of some character strengths compared with those without romantic relationships or living alone, as demonstrated by Karris Bachik et al. (2020) in a sample of college students (those in romantic relationships reported higher scores in the character strengths of love, gratitude, and hope) and by Baumann et al. (2020) in a sample of older adults (those living with a partner reported higher scores mainly in love and teamwork). The character strengths of curiosity, love of learning, perspective, zest, love, kindness, social intelligence, teamwork, self-regulation, gratitude, hope, and humor were found to be negatively related to either attachment avoidance or attachment anxiety, providing indirect support for their relevance in the domain of romantic relationships (Lavy and Littman-Ovadia, 2011). In addition, love, teamwork, fairness, gratitude, and hope were reported to correlate positively with satisfaction with one's family or partnership (Ruch et al., 2010).

Character strengths are also perceived as desirable qualities in romantic partners, which speaks to their relevance in this life domain. Both adolescents (Weber and Ruch, 2012b) and adults (Steen, 2003) value character strengths in potential partners—in particular, the character strengths of honesty, love, kindness, and humor. It also seems that some of the partner's character strengths (perseverance, social intelligence, forgiveness, and prudence) might explain variance in the other partner's life satisfaction beyond the influence of their own character strengths (Weber and Ruch, 2012b). This notion is supported by the finding that both an actor's self-reported strengths endorsement (i.e., the average across all character strengths) and their partner's self-reported strengths endorsement predicted the actor's relationship satisfaction in a sample of married couples (Lavy et al., 2016).

Lavy et al.'s (2016) results also underline the role of strengths-related behavior in romantic relationships: similar to the endorsement of character strengths, both the actor's and the partner's deployment of character strengths (i.e., the extent to which character strengths were shown in the relationship) predicted the actor's relationship satisfaction. While there are, to our knowledge, no published studies that have considered strengths-related behavior at the level of all 24 character strengths, showing gratitude in romantic relationships has also been studied extensively as a powerful predictor of relationship quality and satisfaction (e.g., Algoe et al., 2010).

Hypotheses 8.1–8.9: Building on the results reviewed, we expect the character strengths of honesty, love, kindness, social intelligence, fairness, forgiveness, gratitude, hope, and humor to be of particular relevance in the life domain of romantic relationships (i.e., that they will be perceived as more relevant and displayed more frequently in behavior than in other life domains and that their relevance and display in this life domain will be positively related to flourishing).

## Methods

### Participants

The sample consisted of 203 German-speaking adults (21.2% men, 78.8% women) who were primarily living in Switzerland (66.5%) and Germany (30%). Their mean age was 29.4 years (*SD* = 13.5; ranging from 18 to 77 years). A majority (69.5%) reported being currently in education (school, university, or in-service training; many of which were also working part-time), 26.5% were either employed or self-employed, and 3.0% were currently not in education or working (e.g., unemployed or retired). On average, the sample was highly educated: 57.6% held a higher-education entrance qualification, 26.1% held a university degree, 13.3% had completed vocational training, 1.5% had completed secondary school, and 1.5% were still in secondary school.

The sample size was selected based on considerations regarding statistical power. We wanted to be able to detect a correlation of *r* = 0.30 with a power of at least 0.80 (and an α-level of 0.01 using two-tailed tests). A calculation of the required sample size using G^*^Power 3.1 (Faul et al., 2009) resulted in a sample size of at least *N* = 125. Because participants were able to select the life domains relevant to them (as described in the Procedure section), we recruited more participants with the aim of reaching this target for all of the life domains.

### Instruments

#### Character Strengths

For measuring character strengths, the German version of the VIA-IS (Peterson and Seligman, 2004; German version: Ruch et al., 2010) was used. This instrument consists of 240 items that are rated using a five-point Likert scale (1 = “strongly disagree” to 5 = “strongly agree”), representing the defined 24 character strengths. A sample item for the character strength of gratitude is “I feel thankful for what I have received in life.” Past studies (e.g., Ruch et al., 2010) have provided evidence for the internal consistency (median α = 0.77) and stability (median *r*_tt_ = 0.73 over a period of 9 months) of the VIA-IS. In the present study, the median Cronbach's α was 0.79.

#### Relevance and Strengths-Related Behavior

For measuring the relevance and strengths-related behavior in different life domains, the ACS-RS (Harzer and Ruch, 2013) was used. This instrument measures four aspects of the applicability of each of the 24 character strengths in a certain life domain: (a) promotion, (b) helpfulness, (c) importance, and (d) behavior. For the life domain of work, for example, each character strength is described and rated on these four items: (a) “It is encouraged in my professional life,” (b) “It is helpful in my professional life,” (c) “It is important to me in my professional life,” and (d) “I behave like this in my professional life”. For each life domain, 96 items are rated using a five-point Likert scale (1 = “never” to 5 = “almost always”). The internal consistency of the ACS-RS has been acceptable in earlier studies (Cronbach's α between 0.77 and 0.93). In the current study, each scale was split up into *relevance*, that is, items (a), (b), and (c) and *strengths-related behavior*, that is, item (d). The median Cronbach's alphas for *relevance* in the respective life domain in this study were between 0.78 (education) and 0.89 (leisure).

#### Flourishing

For measuring flourishing, the German version of the Flourishing Scale (FS; Diener et al., 2010; German version: Esch et al., 2013) was used. This instrument consists of eight items rated on a seven-point Likert scale (1 = “strongly disagree” to 7 = “strongly agree”) and covers different aspects considering important characteristics of positive functioning. A sample item is “I am engaged and interested in my daily activities.” In previous studies, this scale has shown a high reliability (Cronbach's α between 0.79 and 0.85). In this study, it yielded an internal consistency of α = 0.87.

### Procedure

According to the guidelines of the institutional ethics board at the University of Zurich, the present study did not require ethical approval. All participants were recruited via university mailing lists, social media, and personal contacts. They participated voluntarily and provided written informed consent. As an incentive for participation, individualized feedback on the individual rank order of character strengths and partial course credit (for students) was offered.

Participants first completed information on demographic variables, followed by the FS, the VIA-IS, and other measures not relevant to the present study. Then, participants were presented with five life domains (work, education, leisure, close personal relationships, and romantic relationships). To enable a common understanding of the life domains, each domain was described briefly. For instance, close personal relationships were described as follows: “Close personal relationships: This life domain includes your family and friends. When answering the following questions, please think of the people with whom you share your thoughts and feelings and with whom you feel closely connected.” The life domain descriptions are provided in the Supplementary Materials. After reading each of the descriptions, participants had to indicate whether this life domain was relevant to them (“Is this life domain a part of your life?”). Participants selected an average of *M* = 4.23 life domains as relevant. Following this selection, they completed the ACS-RS for all the life domains selected. For instance, if a participant indicated that work, leisure, close personal relationships, and romantic relationships were relevant in their life, they completed the ACS-RS four times, once for each of the four domains.

The data were collected as part of a larger project and partly overlap with the sample of self-raters in Study 2 of Wagner et al. (2020a), which studies the relationships between character strengths and orientations to well-being (i.e., PERMA, Seligman, 2011), and one of the four samples of Ruch et al. (2020), which studies the relationships between character strengths and virtues. However, the research questions are unrelated, and the overlap in the data only refers to the VIA-IS.

### Data Analysis

The data analysis followed three steps. To address Hypotheses 1.1–1.11 and Hypotheses 2.1–2.11, both VIA-IS scales and strengths-related behavior (averaged across all relevant life domains) were correlated with flourishing. To determine the amount of variance explained in flourishing by both sets of predictors (addressing Hypothesis 3), we conducted a commonality analysis for each character strength (see, e.g., Nimon and Reio, 2011). This adecomposes the amount of explained variance into variance associated with each predictor uniquely and variance associated with the common effects of all predictors in a multiple regression framework. To conduct the commonality analyses, we performed a set of multiple regression analyses. To address Hypotheses 4.1–4.6, 5.1–5.4, 6.1–6.6, 7.1–7.10, and 8.1–8.9, we conducted two analyses for each life domain: first, *t*-tests were performed for each character strength to compare each life domain's mean on relevance and strengths related-behavior to the respective overall mean across all life domains (e.g., the relevance of creativity at work was compared with the mean relevance of creativity across all five life domains). This approach was chosen because participants were allowed to choose the life domains that they considered important in their lives, and only a smaller subsample of participants selected all domains, making direct comparisons between life domains more difficult. Second, both relevance and strengths-related behavior in each of the life domains were correlated with flourishing. We used the guidelines by Gignac and Szodorai (2016) for research on individual differences to interpret the size of the effects (i.e., *r* = 0.10 representing a small effect, *r* = 0.20 a medium-sized effect, and *r* = 0.30 a large effect). To adjust for the effects of multiple comparisons, we used an α level of 0.01 throughout the analyses.

## Results

Descriptive statistics for the character strengths scales, internal consistency coefficients, and correlations with age and sex are displayed in Supplementary Table 1. Correlations with demographic variables were of small to medium size. Supplementary Tables 2, 3 show the descriptive statistics of the ACS-RS for each of the life domains, separately for relevance and strengths-related behavior.

As shown in Table 1, the highest correlations with flourishing were observed for the character strengths of hope, zest, love, curiosity, perseverance, self-regulation, and teamwork, but all hypothesized character strengths (including honesty, kindness, social intelligence, gratitude, and humor) showed positive correlations with flourishing of at least medium size. Besides the character strengths hypothesized, perspective, bravery, leadership, forgiveness, appreciation of beauty and excellence, and spirituality also showed positive correlations, although of smaller size.

**Table 1:** Correlations between character strengths (VIA-IS scales), strengths-related behavior (mean across all life domains), and flourishing and results of commonality analyses.

	**Correlations with flourishing**		**Amount of explained variance in regression analysis predicting flourishing**			
	**VIA-IS**	**Behavior**	**Unique VIA-IS**	**Unique behavior**	**Common**	**Total**
Creativity	0.17	0.26^*^	0.000	0.041	0.027	0.068
Curiosity	0.44^*^	0.16	0.169	0.002	0.023	0.194
Judgment	0.17	0.23^*^	0.005	0.026	0.025	0.056
Love of learning	0.18	0.12	0.018	0.002	0.013	0.033
Perspective	0.31^*^	0.25^*^	0.048	0.016	0.045	0.109
Bravery	0.21^*^	0.13	0.030	0.002	0.014	0.046
Perseverance	0.44^*^	0.28^*^	0.118	0.002	0.075	0.195
Honesty	0.25^*^	0.24^*^	0.031	0.026	0.031	0.088
Zest	0.61^*^	0.49^*^	0.153	0.025	0.213	0.391
Love	0.55^*^	0.36^*^	0.176	0.004	0.122	0.302
Kindness	0.29^*^	0.31^*^	0.031	0.044	0.051	0.126
Social intelligence	0.38^*^	0.29^*^	0.079	0.015	0.067	0.161
Teamwork	0.40^*^	0.33^*^	0.068	0.005	0.104	0.177
Fairness	0.15	0.26^*^	0.000	0.046	0.022	0.068
Leadership	0.30^*^	0.29^*^	0.029	0.023	0.060	0.112
Forgiveness	0.25^*^	0.17	0.033	0.002	0.028	0.063
Humility	0.07	0.01	0.008	0.003	−0.003	0.008
Prudence	0.14	0.01	0.026	0.005	−0.005	0.026
Self-regulation	0.42^*^	0.16	0.148	0.001	0.024	0.173
Beauty	0.19^*^	0.26^*^	0.002	0.032	0.034	0.068
Gratitude	0.36^*^	0.29^*^	0.053	0.003	0.080	0.136
Hope	0.64^*^	0.36^*^	0.278	0.003	0.125	0.406
Humor	0.31^*^	0.16	0.076	0.005	0.022	0.103
Spirituality	0.19^*^	0.04	0.067	0.031	−0.029	0.069

*N = 203. Beauty = appreciation of beauty and excellence*.

* *p < 0.01 (two-tailed)*.

Strengths-related behavior averaged across life domains was most strongly related to flourishing for the character strengths of zest, hope, love, teamwork, and kindness, but, as hypothesized, also for perseverance, honesty, social intelligence, and gratitude. However, contrary to expectations, no relationships were found for curiosity, self-regulation, and humor.

Different patterns of results could be observed from the commonality analyses (see Table 1). One group of character strengths (curiosity, self-regulation, and humor) showed mostly unique contributions of the VIA-IS scales to the variance explained in flourishing, no unique contributions of strengths-related behavior, and only small contributions of common variance. Another group (perspective, perseverance, zest, love, social intelligence, teamwork, gratitude, and hope) showed mostly unique contributions of the VIA-IS scales and common variance. A third group (honesty, kindness, and leadership) showed relatively equal contributions of all three sources of variance. Finally, a fourth group (creativity, judgment, fairness, and appreciation of beauty and excellence) showed no unique variance explanation in flourishing by the VIA-IS scales but only unique contributions of strengths-related behavior and common variance between both.

Table 2 shows the means of the character strengths' rated relevance for all life domains individually and averaged across life domains, and Table 3 shows the means of strengths-related behavior for all life domains individually and averaged across life domains. Both tables also indicate the results of the *t*-tests comparing the means in each respective life domain with the overall mean across all life domains. The effect sizes (Cohen's *d*) for these comparisons are provided in Supplementary Table 4. The largest effect sizes, that is, the strongest positive deviations from the overall mean, for character strengths' relevance were found for leadership (life domain of work), love of learning (life domain of education), creativity (life domain of leisure), and love (life domains of close personal relationships and romantic relationships). For strengths-related behavior (see Supplementary Table 4), the strongest deviations from the mean across all life domains were observed for the same strengths, except for the life domain of work (strongest effect for self-regulation), but overall, the effect sizes tended to be smaller than for relevance.

**Table 2:** Means and standard deviations of relevance for all character strengths and means across all life domains.

	**Work (*****N*** **= 154)**		**Education (*****N*** **= 179)**		**Leisure (*****N*** **= 190)**		**Close personal relationships (*****N*** **= 197)**		**Romantic relationships (*****N*** **= 140)**		***M* across all life domains**
	***M***	***SD***	***M***	***SD***	***M***	***SD***	***M***	***SD***	***M***	***SD***	
Creativity	3.52	0.96	3.37	0.92	3.92^a^	0.87	3.35^b^	0.86	3.57	0.89	3.53
Curiosity	3.81	0.78	4.23^a^	0.64	4.01	0.76	3.65^b^	0.76	3.76	0.77	3.90
Judgment	3.95^a^	0.74	4.01^a^	0.74	3.44^b^	0.93	3.76	0.70	3.91	0.69	3.78
Love of learning	3.74	0.87	4.31^a^	0.70	3.85^a^	0.92	3.04^b^	0.91	3.37^b^	0.93	3.65
Perspective	3.78	0.85	3.86	0.81	3.45^b^	0.94	3.86	0.71	3.88^a^	0.72	3.74
Bravery	2.90^b^	1.06	2.76^b^	0.96	3.18	1.03	3.39^a^	0.82	3.65^a^	0.88	3.14
Perseverance	3.90	0.77	4.22^a^	0.65	3.98	0.86	3.59^b^	0.81	4.06	0.77	3.93
Honesty	4.06	0.75	3.57^b^	0.93	3.66^b^	1.03	4.34^a^	0.61	4.44^a^	0.57	3.98
Zest	3.73^b^	0.82	3.52^b^	0.81	4.15^a^	0.70	4.13^a^	0.67	4.13^a^	0.69	3.91
Love	3.22^b^	1.02	2.96^b^	1.00	3.07^b^	1.05	4.40^a^	0.64	4.56^a^	0.53	3.60
Kindness	4.23^a^	0.71	3.62^b^	0.87	3.55^b^	1.04	4.50^a^	0.51	4.51^a^	0.49	4.05
Social intelligence	4.15	0.76	3.86^b^	0.88	3.49^b^	1.09	4.53^a^	0.49	4.54^a^	0.51	4.09
Teamwork	4.02^a^	0.85	3.58^b^	0.90	3.41^b^	1.16	4.06^a^	0.74	4.01^a^	0.86	3.78
Fairness	3.96	0.85	3.49^b^	0.90	3.51^b^	1.12	4.18^a^	0.66	4.12^a^	0.74	3.83
Leadership	3.49^a^	1.01	2.95	0.95	2.87	1.14	3.04	0.98	3.04	1.14	3.03
Forgiveness	3.23	0.97	2.75^b^	0.95	2.93^b^	1.10	3.96^a^	0.72	4.19^a^	0.69	3.38
Humility	3.22	0.84	2.98^b^	0.93	3.13^b^	0.98	3.63^a^	0.70	3.66^a^	0.80	3.32
Prudence	3.48^a^	0.89	3.37	0.87	3.19	0.96	3.09^b^	0.89	3.40	0.95	3.28
Self-regulation	3.93^a^	0.75	3.83^a^	0.78	3.39^b^	1.00	3.48^b^	0.80	3.63	0.89	3.63
Beauty	3.08^b^	1.16	2.77^b^	1.03	3.80^a^	1.03	3.65^a^	0.88	3.95^a^	0.95	3.44
Gratitude	3.26^b^	1.06	2.85^b^	0.99	3.49	1.09	3.98^a^	0.73	4.25^a^	0.65	3.54
Hope	3.39^b^	0.95	3.42^b^	0.92	3.52	0.97	3.80^a^	0.76	4.15^a^	0.66	3.62
Humor	3.74	0.93	3.24^b^	0.95	3.62	1.04	4.25^a^	0.65	4.25^a^	0.67	3.78
Spirituality	1.92	1.13	1.83^b^	1.05	2.16	1.28	2.18	1.22	2.21	1.30	2.06

*Beauty = appreciation of beauty and excellence*.

a *Higher than the mean across all life domains (p < 0.01, two-tailed)*.

b *Lower than the mean across all life domains (p < 0.01, two-tailed)*.

**Table 3:** Means and standard deviations of strengths-related behavior for all character strengths and means across all life domains.

	**Work (*****N*** **= 154)**		**Education (*****N*** **= 179)**		**Leisure (*****N*** **= 190)**		**Close personal relationships (*****N*** **= 197)**		**Romantic relationships (*****N*** **= 140)**		***M* across all life domains**
	***M***	***SD***	***M***	***SD***	***M***	***SD***	***M***	***SD***	***M***	***SD***	
Creativity	3.47	1.04	3.23	1.05	3.76^a^	0.92	3.31	0.99	3.52	0.94	3.42
Curiosity	3.95	0.85	4.12^a^	0.77	4.02	0.81	3.73^b^	0.93	3.78	0.82	3.92
Judgment	3.99^a^	0.71	3.84	0.84	3.49^b^	1.03	3.78	0.82	3.85	0.81	3.76
Love of learning	3.73	1.03	4.01^a^	0.87	3.80^a^	0.97	3.19^b^	1.04	3.36^b^	1.04	3.61
Perspective	3.79	0.91	3.75	0.85	3.46^b^	0.96	3.85	0.79	3.84	0.84	3.71
Bravery	3.15	1.15	2.79^b^	1.05	3.14	1.10	3.36^a^	1.00	3.56^a^	0.98	3.16
Perseverance	3.97	0.88	3.97	0.82	3.92	0.95	3.62^b^	0.93	4.03	0.87	3.87
Honesty	4.18	0.79	3.81^b^	0.92	3.91	0.99	4.25^a^	0.77	4.41^a^	0.73	4.08
Zest	3.72	0.90	3.54^b^	0.94	4.01^a^	0.80	3.93	0.81	3.89	0.77	3.79
Love	3.44	1.09	3.14^b^	1.04	3.27^b^	1.11	4.11^a^	0.87	4.34^a^	0.71	3.61
Kindness	4.38^a^	0.74	4.03^b^	0.84	3.85^b^	1.03	4.47^a^	0.62	4.42^a^	0.62	4.21
Social intelligence	4.28	0.75	3.97	0.85	3.75^b^	1.08	4.39^a^	0.69	4.38^a^	0.66	4.13
Teamwork	4.09^a^	0.81	3.64	0.91	3.56^b^	1.13	3.92	0.87	3.89	0.96	3.79
Fairness	4.13^a^	0.76	3.80	0.90	3.63^b^	1.08	4.10^a^	0.70	4.16^a^	0.71	3.93
Leadership	3.55^a^	1.06	3.05	1.08	2.92	1.17	3.07	1.06	3.11	1.18	3.09
Forgiveness	3.38	0.94	3.20^b^	1.02	3.19^b^	1.13	3.78^a^	0.88	3.93^a^	0.86	3.47
Humility	3.68	0.90	3.60	0.97	3.40^b^	1.07	3.77	0.83	3.70	0.91	3.64
Prudence	3.69^a^	0.95	3.62^a^	0.91	3.23	1.05	3.21^b^	0.98	3.39	1.01	3.41
Self-regulation	4.02^a^	0.79	3.81^a^	0.81	3.46	1.03	3.53	0.88	3.47	0.96	3.64
Beauty	3.31	1.16	3.01^b^	1.13	3.78^a^	1.07	3.68^a^	0.97	3.80^a^	1.01	3.49
Gratitude	3.49	1.06	3.27^b^	1.06	3.62	1.12	3.91^a^	0.81	4.10^a^	0.83	3.65
Hope	3.58	0.99	3.50	0.97	3.63	1.03	3.72	0.87	4.03^a^	0.80	3.66
Humor	3.83	1.00	3.59^b^	1.10	3.84	1.03	4.07^a^	0.85	4.09^a^	0.88	3.85
Spirituality	2.09	1.34	1.88	1.19	2.14	1.36	2.12	1.28	2.19	1.36	2.08

*Beauty = appreciation of beauty and excellence*.

a *Higher than the mean across all life domains (p < 0.01, two-tailed)*.

b *Lower than the mean across all life domains (p < 0.01, two-tailed)*.

**Figure 1:**
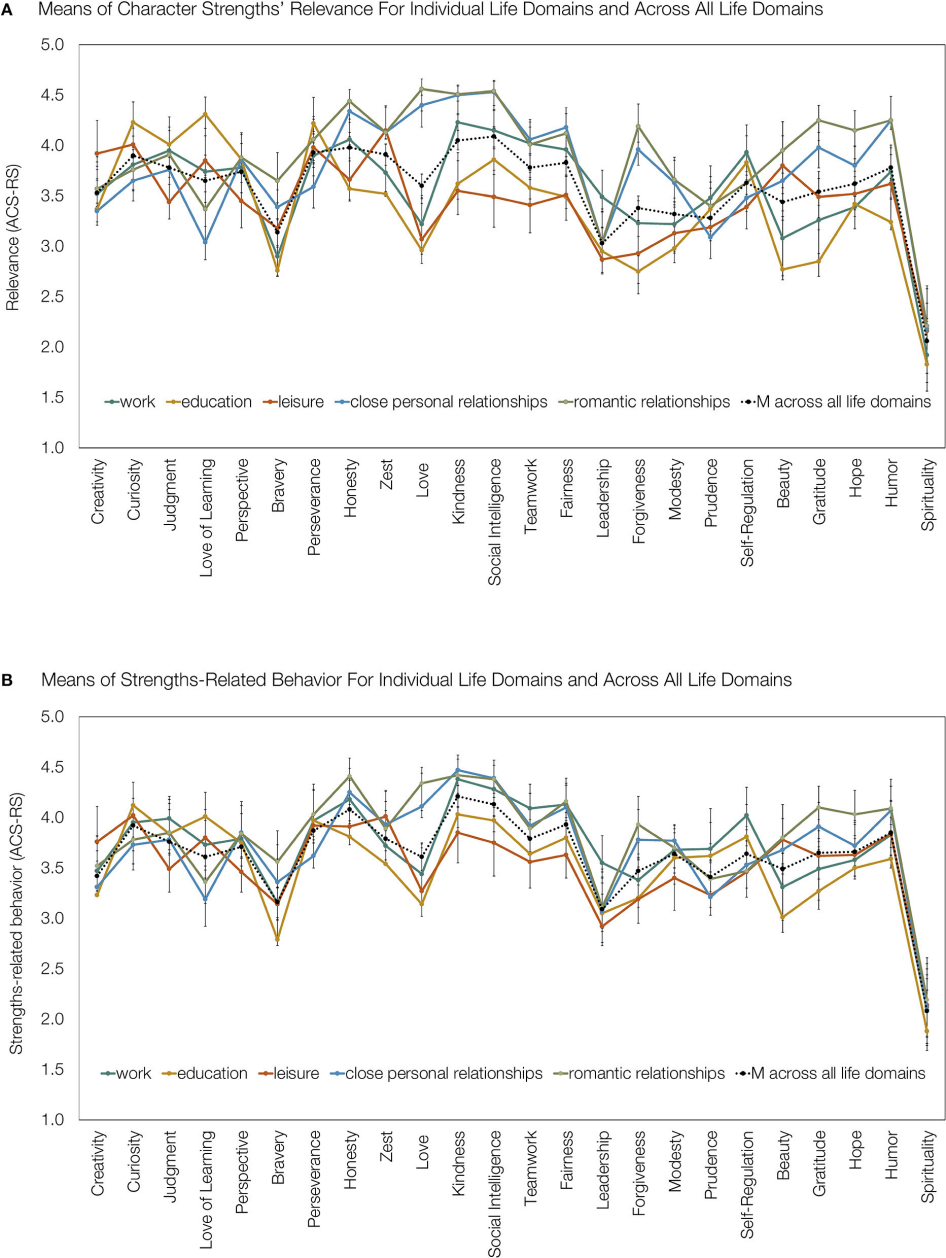
Means of character strengths' relevance **(A)** and strength-related behavior **(B)** with regard to the life domains of work, education, leisure, close personal relationships, romantic relationships, and across all life domains. Beauty = appreciation of beauty and excellence. Error bars: 95% confidence interval. *N* = 140–197.

Overall, the character strengths' relevance and strengths-related behavior showed distinguishable patterns across the different life domains (work, education, leisure, close personal relationships, and romantic relationships). The means are depicted in Figure 1.

As shown in Figure 1, the ratings were most similar for the life domains of close personal relationships and romantic relationships, and the life domains of work and education also showed some overlap but clear differences as well. The domain of leisure showed the fewest similarities with other life domains.

Finally, we analyzed the correlations between the character strengths' relevance and strengths-related behavior for each of the five life domains with flourishing (see Table 4). All effect sizes ranged between medium-sized and large effects. Notably, across all correlations in Table 4, no negative correlation reached statistical significance.

**Table 4:** Correlations of relevance of character strengths and strengths-related behavior across life domains with flourishing.

	**Work (*****N*** **= 154)**		**Education (*****N*** **= 179)**		**Leisure (*****N*** **= 190)**		**Close personal relationships (*****N*** **= 197)**		**Romantic relationships (*****N*** **= 140)**	
	**Relevance**	**Behavior**	**Relevance**	**Behavior**	**Relevance**	**Behavior**	**Relevance**	**Behavior**	**Relevance**	**Behavior**
Creativity	0.16	0.18	0.30^*^	0.29^*^	0.04	0.07	0.12	0.21^*^	0.18	0.26^*^
Curiosity	0.20	0.22^*^	0.20^*^	0.19^*^	0.03	0.05	0.08	0.04	0.11	0.20
Judgment	0.21^*^	0.15	0.24^*^	0.30^*^	0.13	0.09	0.05	0.09	0.05	0.13
Love of learning	0.15	0.16	0.22^*^	0.24^*^	−0.03	−0.06	0.11	0.03	0.11	0.20
Perspective	0.24^*^	0.18	0.19	0.29^*^	0.04	0.09	0.16	0.09	0.15	0.20
Bravery	0.09	0.12	0.13	0.09	0.11	0.04	0.07	0.15	0.02	−0.01
Perseverance	0.17	0.19	0.20^*^	0.25^*^	0.03	0.14	0.17	0.18	0.05	0.04
Honesty	0.24^*^	0.10	0.19	0.20^*^	0.17	0.20^*^	0.15	0.17	−0.10	−0.05
Zest	0.37^*^	0.42^*^	0.37^*^	0.46^*^	0.23^*^	0.27^*^	0.30^*^	0.35^*^	0.18	0.28^*^
Love	0.19	0.09	0.23^*^	0.32^*^	0.29^*^	0.24^*^	0.14	0.20^*^	−0.04	0.03
Kindness	0.19	0.17	0.19	0.25^*^	0.20^*^	0.21^*^	0.13	0.16	0.16	0.26^*^
Social intelligence	0.21^*^	0.15	0.15	0.21^*^	0.21^*^	0.18	0.17	0.16	0.15	0.16
Teamwork	0.27^*^	0.18	0.16	0.21^*^	0.25^*^	0.17	0.21^*^	0.29^*^	0.22^*^	0.21
Fairness	0.26^*^	0.07	0.23^*^	0.21^*^	0.16	0.14	0.13	0.18	0.18	0.13
Leadership	0.24^*^	0.19	0.32^*^	0.24^*^	0.24^*^	0.19^*^	0.26^*^	0.23^*^	0.19	0.13
Forgiveness	0.20	0.13	0.14	0.10	0.21^*^	0.09	−0.01	0.12	0.00	0.08
Humility	0.15	−0.02	0.14	0.04	0.06	−0.01	0.07	0.01	0.01	−0.08
Prudence	0.10	0.04	0.04	0.01	0.09	0.05	0.02	−0.03	−0.01	−0.02
Self-regulation	0.06	0.12	0.07	0.18	0.11	0.10	0.00	0.06	0.05	0.11
Beauty	0.15	0.19	0.24^*^	0.22^*^	0.07	0.11	0.22^*^	0.15	0.08	0.25^*^
Gratitude	0.21^*^	0.29^*^	0.20^*^	0.20^*^	0.14	0.13	0.16	0.17	0.02	0.29^*^
Hope	0.21^*^	0.23^*^	0.21^*^	0.31^*^	0.17	0.11	0.18	0.27^*^	0.01	0.29^*^
Humor	0.10	0.14	0.18	0.12	0.20^*^	0.12	0.10	0.13	0.01	0.11
Spirituality	0.08	0.18	0.04	0.04	−0.05	−0.06	0.00	0.04	0.05	0.03

*Beauty = appreciation of beauty and excellence*.

* *p < 0.01 (two-tailed)*.

## Discussion

The present study investigates how the relevance of character strengths and the frequency of strengths-related behavior differ across life domains and how both relate to overall flourishing. Taken together, the findings demonstrate that different life domains (work, education, leisure, close personal relationships, and romantic relationships) show distinguishable profiles of relevant character strengths. Moreover, strengths-related behavior across different life domains explained additional variance in flourishing beyond the trait level of each respective character strength for a number of character strengths.

The correlations with flourishing were in line with our expectations (Hypotheses 1.1–1.11), whereas additional strong relationships (*r* ≥ 0.30) were observed for perspective, teamwork, and leadership. For most of the hypothesized character strengths (Hypotheses 2.1–2.11), we also found positive relationships of strengths-related behavior across life domains, with the exceptions of curiosity, self-regulation, and humor. While the overall pattern of correlations with flourishing was similar for character strengths (as assessed by the VIA-IS) and averaged strengths-related behavior (as assessed by the ACS-RS) across life domains, some notable differences emerged, which were supported by the results of the commonality analyses. In particular, the character strengths of creativity, judgment, and fairness showed relatively strong contributions of unique variance of strengths-related behavior to flourishing, whereas the VIA-IS scales contributed no unique variance explanation. Conversely, the character strengths of curiosity, self-regulation, and humor showed a strong unique contribution of the VIA-IS scales but no to small contributions of unique variance in strengths-related behavior or common variance, suggesting for these character strengths that aspects other than displaying strengths-related behavior across different life domains are relevant to the strengths' relationships with flourishing. In the case of self-regulation, for instance, it is conceivable that its relationship with health and health behaviors (see Proyer et al., 2013) is more relevant in explaining variance in flourishing than the frequency with which self-regulation is shown in the life domains considered here.

Regarding Hypothesis 3, we found a unique variance explanation in flourishing (of at least *R*^2^ = 0.02) of strengths-related behavior for the character strengths of creativity, judgment, honesty, zest, kindness, fairness, leadership, and appreciation of beauty and excellence and therefore consider the hypothesis supported for these character strengths. For 18 character strengths, the VIA-IS scales and strengths-related behavior jointly explained a small but relevant proportion of the variance of flourishing (with a contribution of common variance of at least *R*^2^ = 0.02), further supporting the role of strengths-related behavior across life domains in explaining variance in flourishing.

**Figure 2:**
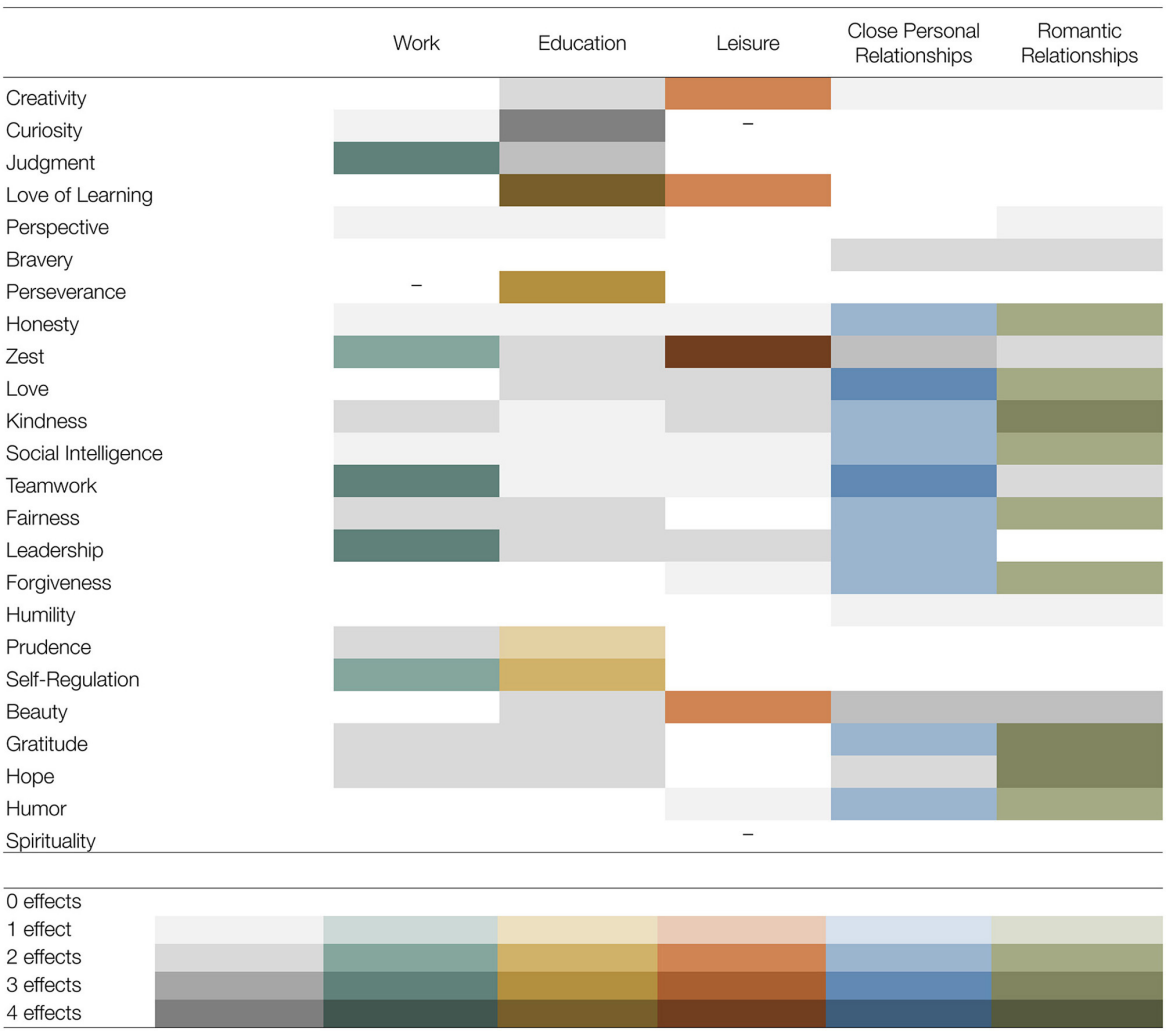
Overview of results regarding the hypotheses on the different life domains. Beauty = appreciation of beauty and excellence. Gray = Effects that were not hypothesized. Colored = Effects that were hypothesized. Different shade of a color denote a different number of effects observed. - = Effects hypothesized, but no effects found.

The findings regarding the different life domains are summarized in Figure 2. It shows for each character strength and life domain how many effects–out of a maximum of four: (1) it was perceived as more relevant than the mean across the life domains, (2) it was displayed more frequently than the mean across the life domains, (3) the relevance in the life domain was related to flourishing, and (4) the display of strengths-related behavior in the life domain was related to flourishing–were found and whether these effects had been hypothesized.

For the life domain of *work*, in line with our expectations (Hypotheses 4.1, 4.4, 4.5, and 4.6), we found evidence for the particular relevance of the character strengths of judgment, teamwork, leadership, and self-regulation; all strengths were perceived as more relevant and displayed more frequently than in other life domains, and their perceived relevance at work was associated with flourishing (with the except for self-regulation). Regarding the character strengths of perseverance (4.2) and zest (4.3), we found no or little support for their particular relevance when displayed in the domain of work, which seems to contradict previous studies highlighting the role of these two character strengths at studies in particular (Peterson et al., 2009; Littman-Ovadia and Lavy, 2016). However, perceiving zest as relevant at work was positively related to flourishing even when controlling for the trait level of zest (additional analyses, not reported in detail here), which might speak to its relevance in this domain. In addition, both character strengths showed relatively little variation and relatively high overall ratings, making it more difficult to demonstrate a higher relevance and frequency of display as compared with other life domains. In conclusion, the present results do not speak against the relevance of perseverance and zest at work, but question whether those character strengths are more relevant in the domain of work than in other life domains. In addition to the character strengths in Hypotheses 4.1–4.6, both kindness and prudence were also perceived as more relevant and displayed more frequently at work than in other life domains.

Regarding *education*, we found support for Hypotheses 5.1 and 5.2 (i.e., for the relevance of love of learning and perseverance); however, perseverance was not displayed more frequently in education than in other life domains. This is in line with studies on the relationships between character strengths and educational outcomes highlighting the important role of these two character strengths in particular (e.g., Wagner and Ruch, 2021). However, less support was found for the relevance of prudence (5.3), which was shown more frequently, and self-regulation (5.4), which was also perceived as more relevant than in other life domains. Additionally, the character strength of judgment was perceived as more relevant here than in other life domains, and its relevance and display in education were related to flourishing. The results also suggested the relevance of curiosity across all four analyses. When considering an average across all the relevance and the display ratings of all 24 character strengths, participants seemed to perceive fewer opportunities to display character strengths in the educational setting than in other life domains, which is also in line with Kachel et al.'s (2020) finding that the applicability of signature strengths in the life domain of education was perceived as lower than that in private life. This might be a starting point for strengths-based interventions in the educational context. Specifically, these might aim to increase awareness of opportunities to display character strengths and to encourage students and staff to create such opportunities.

For the life domain of *leisure*, the character strength of zest was clearly supported as being of particular relevance (Hypothesis 6.4). Three of the remaining five strengths in Hypotheses 6.1–6.6 (creativity, love of learning, and appreciation of beauty and excellence) were also perceived as more relevant and displayed more frequently than in other life domains. However, no support was found for the particular relevance of curiosity (6.2) or spirituality (6.6). Based on our literature review, leisure can be described as an underexplored life domain in terms of the role of character strengths. Given the large variety of leisure activities, future studies might benefit from comparing different types or characteristics of leisure activities with regard to the relevance of character strengths.

Regarding our hypotheses on *close personal relationships* (7.1–7.10), we found the strongest support for the relevance of love, teamwork, and leadership. In addition, honesty, kindness, social intelligence, fairness, forgiveness, and gratitude were also perceived as more relevant and displayed more frequently than in other life domains, so our hypotheses can also be considered confirmed for this set of character strengths.

Finally, for the domain of *romantic relationships* (Hypotheses 8.1–8.9), the most consistent support was observed for the relevance of kindness, gratitude, and hope. Further, honesty, love, social intelligence, fairness, forgiveness, and humor were perceived as more relevant and displayed more frequently than in other life domains. Thus, all these character strengths can also be considered to be of particular relevance in romantic relationships.

When comparing the life domains' profiles, there were strong similarities between the domains of close personal relationships and romantic relationships; the only slight differences come from the character strengths of bravery, perseverance, gratitude, and hope, which were all perceived as somewhat more relevant in the domain of romantic relationships. However, the similarities certainly outweigh the differences, and in future studies aiming to compare life domains, it would be reasonable to merge both types of relationships into one domain. The profiles of the domains of work and education also shared some similarities overall yet were distinguishable by higher levels for curiosity and love of learning in the domain of education and higher levels for character strengths related to interpersonal relationships (e.g., those assigned to the virtues of humanity and justice) in the domain of work.

Overall, some character strengths were considered similarly relevant and displayed similarly frequently across the life domains (e.g., curiosity, spirituality, humility, perspective, perseverance, teamwork, zest, and prudence), whereas these variables varied more strongly between life domains for other character strengths (e.g., love, gratitude, love of learning, appreciation of beauty and excellence, bravery, and forgiveness). The latter set of character strengths might be more sensitive to environmental demands or influences, and these findings may inform the discussion on tonic versus phasic character strengths (see Peterson and Seligman, 2004). Spirituality was found to be least relevant and displayed least frequently in all life domains. However, it also yielded the highest standard deviations in both variables (relevance and strengths-related behavior); that is, a stronger variability between individuals was observed for spirituality than for other character strengths. In addition, some character strengths' relevance ratings displayed little variation between individuals: for instance, love and kindness were rated as highly relevant to close personal relationships and romantic relationships. While this was to be expected, this restricted variation might have impacted the correlations that could be observed with these variables.

### Limitations

Several limitations need to be considered when interpreting the results of the present study. First, the results are based on self-reports, making them prone to potential response biases. However, studies using informant reports of character strengths, well-being or both (e.g., Buschor et al., 2013; Wagner et al., 2020a) have shown that the results are highly comparable with those exclusively using self-reports. Second, we studied five life domains (work, education, leisure, close personal relationships, and romantic relationships) that we considered to be generally most relevant for (young) adults. However, additional or more narrowly defined life domains are conceivable (e.g., volunteer work and parenting/family). In addition, the domains were conceived as broad, general areas of life, and a more fine-grained analysis would certainly warrant further research. For instance, in the domain of education, it would be interesting to investigate to which extent the context of school differs from the context of higher education. As we know from research within individual life domains (e.g., Wagner et al., 2020b), differential relationships of character strengths with specific outcomes within these domains can be anticipated. Because of the study design, which allowed participants to select the life domains that were relevant to their lives and therefore did not require them to answer the questions for all life domains, we were also are not able to directly compare means across the different domains. Third, we only considered relationships to a broad measure of flourishing, and other outcomes (such as other aspects of psychological functioning, achievement, or the well-being of others) would certainly also be of relevance. We also only looked at flourishing in general, not in relation to the specific life domains. It is to be expected that the unique contribution of strengths-related behavior might be even larger if domain-specific outcomes were predicted (see Wagner and Ruch, 2021 for an application in the educational context). Fourth, this study is limited by the composition of the sample: participants were rather young, mostly female, and mostly students or highly educated, which might have led to a biased representation of the life domain of work. In addition, it is conceivable that there are age-related trajectories in the reported associations (see Baumann et al., 2020). Finally, given the cross-sectional nature of the data, the results do not allow for any claims regarding causality or directionality.

### Implications

In general, ratings provided for the relevance of certain character strengths in a specific context may be caused by several factors. The environmental demands or rewards for showing strengths-related behavior are assumed to represent a shared perception by everyone in that environment, which is supported by findings that suggest a considerable agreement between different raters regarding the relevance of character strengths in a given context (Harzer and Ruch, 2013). However, an individual's perception of opportunities to display a certain strength is by no means unrelated to the individual's level of character strengths and flourishing: generally, individuals high in a certain strength also tend to see this strength as more relevant (see Supplementary Table 5). As a consequence, when aiming to increase the relevance of character strengths in a certain life domain or environment, both the objective environment and the individual's perception of opportunities to display certain character strengths can be targeted (see job crafting toward strengths; Kooij et al., 2017).

It seems that individuals perceive opportunities to display a larger number of character strengths in the domains of close personal relationships and romantic relationships. Thus, these life domains may also be promising starting points in character strengths-based interventions. More generally, the present results inform character strengths-based interventions on the general patterns of relevance in life domains, which may be used in the design of interventions.

The present study's results also trigger open questions to be addressed in future research. Such open questions include: is it relevant in how many life domains an individual perceives a certain character strength as relevant or displays a character strength? Are there compensation effects—that is, if a character strength is considered to be of low relevance in one domain (such as work), is it more frequently displayed in another domain (such as leisure) as a consequence?

Moreover, the present study also has implications for the study of character strengths in general: we were able to demonstrate that the average strengths-related behavior across different life domains was, in some cases, a better predictor of flourishing than the respective VIA-IS scale. This suggests that the relationships of some character strengths, such as creativity, judgment, and fairness, to flourishing might have been somewhat underestimated in previous research using the VIA-IS. In revising the VIA-IS or in constructing other measures of character strengths, it would be advisable to consider the item content carefully with regard to the representation of items relating to affect, behavior, cognition, and desire (ABCD; Wilt and Revelle, 2015).

## Conclusions

The literature review revealed that there is a relative lack of knowledge regarding the role of character strengths in several life domains, in particular, adult romantic relationships and close personal relationships, and leisure. Future research programs might be devoted to shedding more light on character strengths' contribution to flourishing in these life domains. The present study underlines that studying the role of character strengths in different life domains allows for more nuanced conclusions than only relying on the trait levels of character strengths.

## Data Availability Statement

The dataset underlying this article is available on the Open Science Framework: https://osf.io/7zvgq/.

## Ethics Statement

Ethical review and approval was not required for the study on human participants in accordance with the local legislation and institutional requirements. The participants provided their written informed consent to participate in this study.

## Author Contributions

LW and WR designed the study. LW oversaw data collection and collected parts of the data. LW and LP analyzed the data and contributed to writing the manuscript. All authors provided feedback and approved the final version.

## Conflict of Interest

WR is a Senior Scientist for the VIA Institute on Character, which holds the copyright to the VIA Inventory of Strengths. The remaining authors declare that the research was conducted in the absence of any commercial or financial relationships that could be construed as a potential conflict of interest.
