# Supplementary material for: Character Strengths in the Life Domains of Work, Education, Leisure, and Relationships and Their Associations With Flourishing
Source: Front Psychol. 2021 Apr 21;12:597534. doi: 10.3389/fpsyg.2021.597534 (PMC8096931; doi:10.3389/fpsyg.2021.597534)
Supplement: Supplementary file 1 [file Data_Sheet_1.pdf]

Supplementary Materials for ”Character Strengths in the Life Domains of Work, Education, Leisure, and Relationships, and their Associations with Flourishing”

(1) Life Domain Descriptions

German:

Beruf:
Bei diesem Lebensbereich handelt es sich um jede Art von Arbeitsstelle. Es kann sich um einen Nebenjob, eine Teilzeit- oder Vollzeitstelle handeln, um ein Angestelltenverhältnis oder eine selbstständige Tätigkeit. Wenn Sie verschiedenen beruflichen Tätigkeiten nachgehen, dann denken Sie bitte bei Beantwortung der folgenden Fragen an die Tätigkeiten, die Ihnen am wichtigsten ist.

Ausbildung:
Bei diesem Lebensbereich handelt es sich um jede mögliche Art von Ausbildung: Schule, Studium, Weiterbildungsmaßnahmen außerhalb der Arbeitszeit und auch autodidaktisches Lernen z.B. von Sprachen. Sollten Sie mehrere Arten von Ausbildung ausüben, dann denken Sie bei der Beantwortung der folgenden Fragen bitte an die Ausbildung, die Ihnen am wichtigsten ist.

Enges soziales Umfeld:
Dies schließt Ihre Familie und Freunde ein. Bitte denken Sie bei der Beantwortung der folgenden Fragen an die Menschen, mit denen Sie Ihre Gedanken und Gefühle teilen und mit denen Sie sich eng verbunden fühlen.

Partnerschaft:
Partnerschaft mit einem Lebenspartner oder einer Lebenspartnerin, Freund / Freundin, Ehemann / Ehefrau etc.

Freizeitbeschäftigung:
Bei diesem Lebensbereich handelt es sich um jede Art von Freizeitbeschäftigung, wie zum Beispiel sportliche (z.B. Klettern), religiöse (z.B. Gottesdienst), ehrenamtliche (z.B. Ferienfahrten mit Kindern) oder kreative (z.B. Kochen) Beschäftigungen. Wenn Sie verschiedenen Freizeitbeschäftigungen nachgehen, dann denken Sie bitte an die Freizeitbeschäftigung, die Ihnen am wichtigsten ist.

English (tentative translations):

Work:
This area of life refers to any type of work. It can be a part-time job, a part-time or full-time job, an employee or self-employed. If you have more than one job, please think of the occupation that is most important to you when answering the following questions.

Education:
This area of your life refers to any type of training: school, university, further education outside working hours, and also self-taught learning of languages, for example. If you pursue more than one type of education, please think of the education that is most important to you when answering the following questions.

Close personal relationships:
This includes your family and friends. When answering the following questions, please think about the people with whom you share your thoughts and feelings and with whom you feel a close connection.

Romantic relationships:
Partnership with a life partner, boyfriend/girlfriend, husband/wife, etc.

Leisure:
This life domain is about any type of leisure activity, such as athletic (e.g., rock climbing), religious (e.g., worship), volunteer (e.g., vacation trips with children), or creative (e.g., cooking) pursuits. If you pursue more than one leisure activity, please think of the leisure activity that is most important to you.

(2) Supplementary tables

Table S1

Means, standard deviations, internal consistency coefficients, correlations with age and sex of all VIA-IS scales

	M	*SD*	α	r_{age}	r_{sex}
Creativity	3.40	0.68	.89	.16*	-.20*
Curiosity	3.82	0.50	.78	.37*	-.15*
Judgment	3.88	0.49	.79	.05	-.08
Love of Learning	3.61	0.60	.81	.17*	-.04
Perspective	3.65	0.50	.80	-.08	-.09
Bravery	3.52	0.55	.78	.25*	-.13
Perseverance	3.50	0.61	.86	.14*	-.07
Honesty	3.84	0.45	.75	.24*	.00
Zest	3.43	0.59	.81	.17*	-.14
Love	3.93	0.56	.78	.17*	-.06
Kindness	3.82	0.47	.73	.20*	-.03
Social Intelligence	3.81	0.46	.78	.02	-.07
Teamwork	3.66	0.49	.75	.15*	-.17*
Fairness	3.88	0.48	.76	.23*	.03
Leadership	3.63	0.47	.74	.18*	-.09
Forgiveness	3.40	0.56	.80	.29*	-.04
Humility	3.21	0.59	.81	.15*	.10
Prudence	3.44	0.59	.80	.11	-.01
Self-Regulation	3.20	0.59	.74	.18*	-.18*
Beauty	3.41	0.61	.78	.12	.07
Gratitude	3.71	0.55	.82	.17*	.15*
Hope	3.55	0.59	.79	.22*	-.08
Humor	3.59	0.61	.87	.02	-.15*
Spirituality	2.54	0.92	.91	.22*	.15*

Notes. N = 203. Beauty = appreciation of beauty and excellence. Age = Age in years. Sex: 1 = male, 2 = female.
*$p < .05$ (two-tailed).

Table S2

Means, Standard Deviations and Internal Consistency Coefficients for Character Strengths' Relevance (ACS-RS) Across all Life Domains

	Work *N* = 154			Education *N* = 179			Leisure *N* = 190			Close Personal Relationships *N* =197			Romantic Relationships *N* = 140		
	M	*SD*	α	*M*	*SD*	α	*M*	*SD*	α	*M*	*SD*	α	*M*	*SD*	α
Creativity	3.52	0.96	.84	3.37	0.92	.81	3.92	0.87	.88	3.35	0.86	.82	3.57	0.89	.83
Curiosity	3.81	0.78	.74	4.23	0.64	.73	4.01	0.76	.84	3.65	0.76	.75	3.76	0.77	.80
Judgment	3.95	0.74	.72	4.01	0.74	.74	3.44	0.93	.84	3.76	0.70	.71	3.91	0.69	.76
Love of Learning	3.74	0.87	.79	4.31	0.70	.75	3.85	0.92	.86	3.04	0.91	.81	3.37	0.93	.87
Perspective	3.78	0.85	.79	3.86	0.81	.76	3.45	0.94	.85	3.86	0.71	.75	3.88	0.72	.82
Bravery	2.90	1.06	.89	2.76	0.96	.78	3.18	1.03	.90	3.39	0.82	.81	3.65	0.88	.84
Perseverance	3.90	0.77	.75	4.22	0.65	.60	3.98	0.86	.87	3.59	0.81	.81	4.06	0.77	.83
Honesty	4.06	0.75	.71	3.57	0.93	.76	3.66	1.03	.88	4.34	0.61	.79	4.44	0.57	.74
Zest	3.73	0.82	.71	3.52	0.81	.70	4.15	0.70	.84	4.13	0.67	.80	4.13	0.69	.77
Love	3.22	1.02	.84	2.96	1.00	.78	3.07	1.05	.90	4.40	0.64	.84	4.56	0.53	.74
Kindness	4.23	0.71	.72	3.62	0.87	.77	3.55	1.04	.89	4.50	0.51	.77	4.51	0.49	.72
Social Intelligence	4.15	0.76	.75	3.86	0.88	.78	3.49	1.09	.89	4.53	0.49	.79	4.54	0.51	.79
Teamwork	4.02	0.85	.78	3.58	0.90	.80	3.41	1.16	.92	4.06	0.74	.80	4.01	0.86	.84
Fairness	3.96	0.85	.77	3.49	0.90	.76	3.51	1.12	.91	4.18	0.66	.81	4.12	0.74	.80
Leadership	3.49	1.01	.81	2.95	0.95	.80	2.87	1.14	.90	3.04	0.98	.86	3.04	1.14	.91
Forgiveness	3.23	0.97	.82	2.75	0.95	.79	2.93	1.10	.89	3.96	0.72	.76	4.19	0.69	.80
Humility	3.22	0.84	.72	2.98	0.93	.75	3.13	0.98	.85	3.63	0.70	.72	3.66	0.80	.80
Prudence	3.48	0.89	.82	3.37	0.87	.82	3.19	0.96	.87	3.09	0.89	.85	3.40	0.95	.86
Self-Regulation	3.93	0.75	.71	3.83	0.78	.72	3.39	1.00	.89	3.48	0.80	.82	3.63	0.89	.84
Beauty	3.08	1.16	.85	2.77	1.03	.82	3.80	1.03	.91	3.65	0.88	.83	3.95	0.95	.89
Gratitude	3.26	1.06	.83	2.85	0.99	.80	3.49	1.09	.91	3.98	0.73	.82	4.25	0.65	.78
Hope	3.39	0.95	.79	3.42	0.92	.78	3.52	0.97	.84	3.80	0.76	.82	4.15	0.66	.82
Humor	3.74	0.93	.82	3.24	0.95	.82	3.62	1.04	.88	4.25	0.65	.79	4.25	0.67	.83
Spirituality	1.92	1.13	.88	1.83	1.05	.87	2.16	1.28	.95	2.18	1.22	.93	2.21	1.30	.93

Notes. Beauty = appreciation of beauty and excellence.

Table S3

Means and Standard Deviations for Strengths-Related Behavior (ACS-RS) Across all Life Domains

	Work<br>*N* = 154		Education<br>*N* = 179		Leisure<br>*N* = 190		Close Personal Relationships<br>*N* = 197		Romantic Relationships<br>*N* = 140	
	M	*SD*	*M*	*SD*	*M*	*SD*	*M*	*SD*	*M*	*SD*
Creativity	3.47	1.04	3.23	1.05	3.76	0.92	3.31	0.99	3.52	0.94
Curiosity	3.95	0.85	4.12	0.77	4.02	0.81	3.73	0.93	3.78	0.82
Judgment	3.99	0.71	3.84	0.84	3.49	1.03	3.78	0.82	3.85	0.81
Love of Learning	3.73	1.03	4.01	0.87	3.80	0.97	3.19	1.04	3.36	1.04
Perspective	3.79	0.91	3.75	0.85	3.46	0.96	3.85	0.79	3.84	0.84
Bravery	3.15	1.15	2.79	1.05	3.14	1.10	3.36	1.00	3.56	0.98
Perseverance	3.97	0.88	3.97	0.82	3.92	0.95	3.62	0.93	4.03	0.87
Honesty	4.18	0.79	3.81	0.92	3.91	0.99	4.25	0.77	4.41	0.73
Zest	3.72	0.90	3.54	0.94	4.01	0.80	3.93	0.81	3.89	0.77
Love	3.44	1.09	3.14	1.04	3.27	1.11	4.11	0.87	4.34	0.71
Kindness	4.38	0.74	4.03	0.84	3.85	1.03	4.47	0.62	4.42	0.62
Social Intelligence	4.28	0.75	3.97	0.85	3.75	1.08	4.39	0.69	4.38	0.66
Teamwork	4.09	0.81	3.64	0.91	3.56	1.13	3.92	0.87	3.89	0.96
Fairness	4.13	0.76	3.80	0.90	3.63	1.08	4.10	0.70	4.16	0.71
Leadership	3.55	1.06	3.05	1.08	2.92	1.17	3.07	1.06	3.11	1.18
Forgiveness	3.38	0.94	3.20	1.02	3.19	1.13	3.78	0.88	3.93	0.86
Humility	3.68	0.90	3.60	0.97	3.40	1.07	3.77	0.83	3.70	0.91
Prudence	3.69	0.95	3.62	0.91	3.23	1.05	3.21	0.98	3.39	1.01
Self-Regulation	4.02	0.79	3.81	0.81	3.46	1.03	3.53	0.88	3.47	0.96
Beauty	3.31	1.16	3.01	1.13	3.78	1.07	3.68	0.97	3.80	1.01
Gratitude	3.49	1.06	3.27	1.06	3.62	1.12	3.91	0.81	4.10	0.83
Hope	3.58	0.99	3.50	0.97	3.63	1.03	3.72	0.87	4.03	0.80
Humor	3.83	1.00	3.59	1.10	3.84	1.03	4.07	0.85	4.09	0.88
Spirituality	2.09	1.34	1.88	1.19	2.14	1.36	2.12	1.28	2.19	1.36

Notes. Beauty = appreciation of beauty and excellence.

Table S4

Effect sizes (Cohen's d) for Comparisons of Respective Life Domain with the Mean Across all Life Domains

	Character Strengths' Relevance					Strengths-Related Behavior				
	Work N = 154	Education N = 179	Leisure N = 190	Close Personal Relationships N = 197	Romantic Relationships N = 140	Work N = 154	Education N = 179	Leisure N = 190	Close Personal Relationships N = 197	Romantic Relationships N = 140
Creativity	-0.01	-0.17	0.45	-0.21	0.04	0.05	-0.18	0.37	-0.11	0.11
Curiosity	-0.12	0.52	0.14	-0.33	-0.18	0.04	0.26	0.12	-0.20	-0.17
Judgment	0.23	0.31	-0.37	-0.03	0.19	0.32	0.10	-0.26	0.02	0.11
Love of Learning	0.10	0.94	0.22	-0.67	-0.30	0.12	0.46	0.20	-0.40	-0.24
Perspective	0.05	0.15	-0.31	0.17	0.19	0.09	0.05	-0.26	0.18	0.15
Bravery	-0.23	-0.40	0.04	0.30	0.58	-0.01	-0.35	-0.02	0.20	0.41
Perseverance	-0.04	0.45	0.06	-0.42	0.17	0.11	0.12	0.05	-0.27	0.18
Honesty	0.11	-0.44	-0.31	0.59	0.81	0.13	-0.29	-0.17	0.22	0.45
Zest	-0.22	-0.48	0.34	0.33	0.32	-0.08	-0.27	0.28	0.17	0.13
Love	-0.37	-0.64	-0.50	1.25	1.81	-0.16	-0.45	-0.31	0.57	1.03
Kindness	0.25	-0.49	-0.48	0.88	0.94	0.23	-0.21	-0.35	0.42	0.34
Social Intelligence	0.08	-0.26	-0.55	0.90	0.88	0.20	-0.19	-0.35	0.38	0.38
Teamwork	0.28	-0.22	-0.32	0.38	0.27	0.37	-0.16	-0.20	0.15	0.10
Fairness	0.15	-0.38	-0.29	0.53	0.39	0.26	-0.14	-0.28	0.24	0.32
Leadership	0.46	-0.08	-0.14	0.01	0.01	0.43	-0.04	-0.15	-0.02	0.02
Forgiveness	-0.15	-0.66	-0.41	0.81	1.17	-0.10	-0.26	-0.25	0.35	0.53
Humility	-0.12	-0.37	-0.19	0.44	0.43	0.04	-0.04	-0.22	0.16	0.07
Prudence	0.22	0.10	-0.09	-0.21	0.13	0.29	0.23	-0.17	-0.20	-0.02
Self-Regulation	0.40	0.26	-0.24	-0.19	0.00	0.48	0.21	-0.17	-0.13	-0.18
Beauty	-0.31	-0.65	0.35	0.24	0.54	-0.16	-0.42	0.27	0.20	0.31
Gratitude	-0.26	-0.70	-0.05	0.60	1.09	-0.15	-0.36	-0.03	0.32	0.54
Hope	-0.24	-0.22	-0.10	0.24	0.80	-0.08	-0.16	-0.03	0.07	0.46
Humor	-0.04	-0.57	-0.15	0.72	0.70	-0.02	-0.24	-0.01	0.26	0.27
Spirituality	-0.12	-0.22	0.08	0.10	0.12	0.01	-0.17	0.04	0.03	0.08

Notes. Beauty = appreciation of beauty and excellence.

Table S5

Correlations of VIA-IS Scales with Character Strengths' Relevance Across Different Life Domains

	Work<br>*N* = 154	Education<br>*N* = 179	Leisure<br>*N* = 190	Close Personal Relationships<br>*N* = 197	Romantic Relationships<br>*N* = 140	Mean Relevance<br>*N* = 203
Creativity	.45*	.53*	.50*	.50*	.51*	.63*
Curiosity	.42*	.18	.36*	.36*	.26*	.44*
Judgment	.27*	.32*	.27*	.16	.16	.35*
Love of Learning	.32*	.28*	.36*	.25*	.10	.42*
Perspective	.35*	.24*	.30*	.40*	.36*	.45*
Bravery	.23*	.17	.26*	.26*	.33*	.33*
Perseverance	.34*	.21*	.26*	.26*	.17	.40*
Honesty	.25*	.32*	.15	.21*	.20	.31*
Zest	.32*	.45*	.32*	.25*	.24*	.46*
Love	.26*	.28*	.40*	.26*	.09	.48*
Kindness	.27*	.29*	.24*	.21*	.25*	.39*
Social Intelligence	.27*	.28*	.23*	.22*	.20	.36*
Teamwork	.43*	.30*	.32*	.42*	.34*	.52*
Fairness	.38*	.38*	.29*	.41*	.30*	.50*
Leadership	.48*	.40*	.32*	.45*	.38*	.53*
Forgiveness	.41*	.25*	.20*	.28*	.12	.38*
Humility	.39*	.43*	.34*	.53*	.36*	.51*
Prudence	.29*	.26*	.28*	.28*	.21	.35*
Self-Regulation	.18	-.01	.20*	.21*	.22	.23*
Beauty	.43*	.48*	.41*	.46*	.53*	.58*
Gratitude	.51*	.44*	.45*	.44*	.41*	.59*
Hope	.29*	.21*	.31*	.21*	.10	.33*
Humor	.54*	.50*	.41*	.52*	.41*	.61*
Spirituality	.65*	.65*	.71*	.75*	.79*	.77*

Notes. Beauty = appreciation of beauty and excellence.
*$p < .01$ (two-tailed).

Table S7

Correlations of VIA-IS Scales with Strengths-Related Behavior Across Different Life Domains

	Work N = 154	Education N = 179	Leisure N = 190	Close Personal Relationships N = 197	Romantic Relationships N = 140	Mean Behavior N = 203
Creativity	.51*	.57*	.48*	.57*	.50*	.67*
Curiosity	.40*	.27*	.36*	.34*	.28*	.45*
Judgment	.36*	.33*	.29*	.34*	.40*	.49*
Love of Learning	.34*	.39*	.40*	.30*	.28*	.49*
Perspective	.22*	.31*	.37*	.23*	.37*	.44*
Bravery	.35*	.26*	.25*	.39*	.41*	.42*
Perseverance	.38*	.50*	.37*	.35*	.21*	.54*
Honesty	.23*	.37*	.18	.28*	.24*	.35*
Zest	.49*	.59*	.33*	.49*	.25*	.60*
Love	.18	.41*	.36*	.46*	.26*	.55*
Kindness	.37*	.27*	.27*	.37*	.31*	.41*
Social Intelligence	.33*	.38*	.24*	.42*	.30*	.46*
Teamwork	.40*	.39*	.32*	.46*	.43*	.57*
Fairness	.50*	.36*	.27*	.48*	.35*	.53*
Leadership	.44*	.43*	.39*	.45*	.30*	.54*
Forgiveness	.55*	.45*	.30*	.46*	.25*	.54*
Humility	.41*	.53*	.50*	.59*	.49*	.66*
Prudence	.43*	.44*	.43*	.42*	.28*	.53*
Self-Regulation	.29*	.21*	.21*	.21*	.31*	.32*
Beauty	.49*	.51*	.40*	.49*	.49*	.60*
Gratitude	.55*	.54*	.44*	.48*	.61*	.68*
Hope	.38*	.40*	.23*	.37*	.42*	.49*
Humor	.67*	.60*	.42*	.59*	.49*	.68*
Spirituality	.65*	.67*	.68*	.72*	.80*	.78*

Notes. Beauty = appreciation of beauty and excellence.
*p < .01 (two-tailed).
